# Charting Morphotoxicity With Complementary Embryo Models

**DOI:** 10.1002/adhm.202503634

**Published:** 2026-08-27

**Authors:** Dorian G. Luijkx, Pauline L. Balder, Romy M. L. Verhagen, Vinidhra Shankar, Barry Jutten, Pinak Samal, Timo Rademakers, Clemens A. van Blitterswijk, Stefan Giselbrecht, Erik J. Vrij

**Affiliations:** ^1^ MERLN Institute for Technology‐Inspired Regenerative Medicine, Department for Cell Biology‐inspired Tissue Engineering Maastricht University Maastricht The Netherlands; ^2^ GROW Research Institute for Oncology and Reproduction, Department of Obstetrics & Gynecology Maastricht University Maastricht The Netherlands; ^3^ MERLN Institute for Technology‐Inspired Regenerative Medicine Department for Complex Tissue Regeneration Maastricht University Maastricht The Netherlands; ^4^ MERLN Institute for Technology‐Inspired Regenerative Medicine Department for Instructive Biomaterials Engineering Maastricht University Maastricht The Netherlands

**Keywords:** blastoids, postimplantation embryo models, stem cell‐based embryo models, thermoformed microwells, toxicity screening

## Abstract

Understanding developmental toxicity demands capturing the full spectrum of morphological diversity, or morphospace, that defines early human embryogenesis. Traditional toxicity testing methods, relying on animal models or simplified 2D cell cultures, inherently overlook the nuanced interplay between distinct developmental stages and cell lineages. Addressing this gap, we present an innovative high‐throughput screening approach leveraging three complementary 3D stem cell‐based embryo models: preimplantation stage blastoids, and early postimplantation stage epiblast‐like (EPICs) and amnion‐like clusters (AMNICs). Cultured in thermoformed microwell arrays, these models were systematically exposed to a library of 27 widely used compounds. Automated image‐based analyses allowed for precise quantification of morphotoxic effects. Key findings include the identification of compounds such as ascorbic acid and valproic acid (VPA), which demonstrated distinct and opposing effects across the different embryo models, highlighting developmental stage‐specific sensitivities. This integrative platform underscores the essential role of complementary embryo models to achieve robust and human‐relevant developmental toxicity assessment.

## Graphical abstract

Three human embryo models of the peri‐implantation stages are exposed to a library of compounds. The (toxic) effects of the compounds are analyzed through automated image analysis pipelines. By evaluating the differential responses of the embryo models, this study underscores the importance of testing complementary embryo models to achieve robust and human‐relevant developmental toxicity assessment.

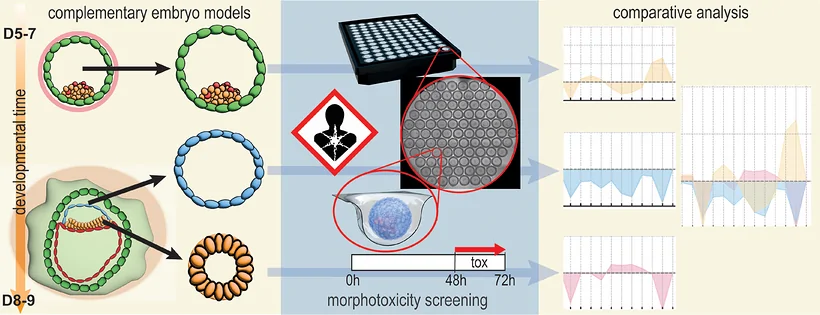

## 1 Introduction

The peri‐implantation and early postimplantation stages of human development represent a critical window during which essential processes occur to sustain further development. During this period, the blastocyst implants into the uterine wall, the epiblast undergoes polarization and cavitation, leading to the formation of the embryonic sac with amnion cells emerging on the dorsal side [1]. Any disruptions at this sensitive stage can have profound effects on embryonic viability and long‐term development [2]. Pregnancy loss is particularly high during this period, with an estimated 60% of all conceptions being lost before clinical pregnancy detection. While pregnancy losses and complications during these early stages can be in part attributed to chromosomal abnormalities, environmental factors including toxicant exposures may also contribute [3, 4, 5]. Therefore, toxicity studies focused on this developmental stage could play a role in preventing infertility.

However, studies on toxicity during peri‐implantation are sparse due to the inaccessibility of natural human embryos in the numbers required for robust toxicity studies [6]. Beyond technical limitations, the use of human embryos for scientific and especially commercial applications comes with significant ethical considerations. As a result, current methods for developmental toxicity screening rely heavily on animal models or simplified in vitro systems [5, 7, 8]. Both approaches have considerable drawbacks of their own. Animal studies are costly, raise ethical concerns, and may not reliably predict effects in human embryos due to species‐specific differences in embryonic development [9, 10]. On the other hand, large scale in vitro assays are often based on 2D human cell cultures, which do not recapitulate the 3D‐complexity of embryonic tissues and is therefore unsuitable for assessing development‐disrupting morphological changes; an effect termed morphotoxicity [11, 12]. This inability to assess how compounds affect the shape, organization, and coordinated growth of tissues represents a major gap in safety assessment.

To bridge this gap, 3D stem cell‐based embryo models are emerging as powerful tools that human‐relevant, scalable, and crucially, enable the quantification of morphological features. By systematically perturbing these models with compound libraries, it becomes possible to map the phenotypic landscape, providing deep insights into the mechanisms underlying developmental toxicity [12, 13, 14, 15]. One common implicit assumption, however, is that models covering similar lineage(s) and/or overlapping developmental time frames are interchangeable. As postulated in the concept of “morphospace”, a space encompassing all possible forms, there is significant added value in exploring a breadth of similar embryo models, as each occupies a unique position and can reveal different developmental constraints [16, 17]. Along this line of thinking, we hypothesize that the use of complementary 3D embryo models for toxicity screening provides a more reliable and accurate picture of toxicity at a particular developmental stage.

We established a toxicity screen of three complementary 3D embryo models of the peri and early postimplantation stage, namely blastoids, epiblast embryoids, and amnion embryoids, to investigate their response to a library of widely used compounds. The blastoid mimics the peri‐implantation human blastocyst [18, 19, 20, 21, 22]. Blastoids contain all three cell types present in natural human blastocysts and are able to mimic numerous processes, from the first lineage segregation to the attachment to the endometrium [18, 19, 20]. In addition to blastoids, epiblast and amnion embryoids (here referred to as epiblast‐like clusters (EPICs) and amnion‐like clusters (AMNICs)) were included in this study as early postimplantation models [15, 23, 24, 25]. In vivo, the epiblast eventually gives rise to the embryo proper, while the amnion contributes to the extraembryonic tissues and plays an important role in early epiblast patterning. The epiblast emerges during preimplantation and then further matures and undergoes lumenogenesis upon implantation. As the lumen arises within the epiblast cluster in vivo, the epiblast cells nearest to the trophectoderm differentiate to amnion cells [15, 23]. EPICs and AMNICs represent these two cell types and their cavitated 3D morphologies during this early stage of postimplantation development, in which the early epiblast forms the pro‐amniotic cavity with squamous amnion cells on the dorsal side, induced by BMPs from the adjacent trophoblasts, and a pseudostratified layer of epiblast cells on the ventral side of the cavity [1, 26]. In vitro, amnion can be derived from primed human embryonic stem cells (hESCs), which are similar to postimplantation epiblast, through induction with BMP4 [15, 24, 27]. EPICs, on the other hand, can be formed by bringing hESCs in suspension culture in medium that maintains their epiblast identity (e.g., Activin‐A and FGF) [28, 29], while this same suspension culture can give rise to amnion‐like cysts by the addition of BMP4 to the medium [24]. Through these separate cultures, the cellular responses of these specific lineages can be decoupled and studied independently, allowing us to compare the effects of compounds on these individual cell types in a 3D context.

Due to the small size of the blastoids, EPICs, and AMNICs (up to 300 µm in diameter), these cultures are all readily assessable through high‐resolution imaging with common fluorescent dyes. Furthermore, by culturing these three embryo models in 96‐wellplate microwell platforms, they can be generated uniformly in a high‐throughput format, enabling the testing of a small library of compounds simultaneously in a single plate. Automated image analysis would further enhance the platform by enabling rapid and objective assessment of morphological and cellular changes. Together, these innovations could offer a robust system for evaluating developmental toxicity in a human‐relevant context.

Here, we establish as a proof‐of‐concept, a high‐throughput morphotoxicity screening platform using human blastoids, EPICs, and AMNICs to evaluate toxicity in complementarity models for the peri‐implantation window of human development. We use a 96‐wellplate‐based thermoformed microwell screening plate platform [30] in conjunction with fluorescent image‐based readouts that are analyzed using the open‐source image analysis software CellProfiler and CellProfiler Analyst [31] to identify compounds with potential disrupting effects on early embryonic processes. This allows for a scalable and objective assessment of toxic effects. Importantly, our results reveal that different toxic compounds exhibit selective disruptive effects depending on embryonic tissue type. With this work, we provide a scalable, integrated, and human‐relevant toxicity screening approach that not only enhances our understanding of developmental perturbations but also paves the way for more accurate risk assessments in both pharmaceutical development and environmental health.

## 2 Results

### 2.1 High‐Throughput Generation of Stem Cell‐Based Embryo Models

**FIGURE 1:**
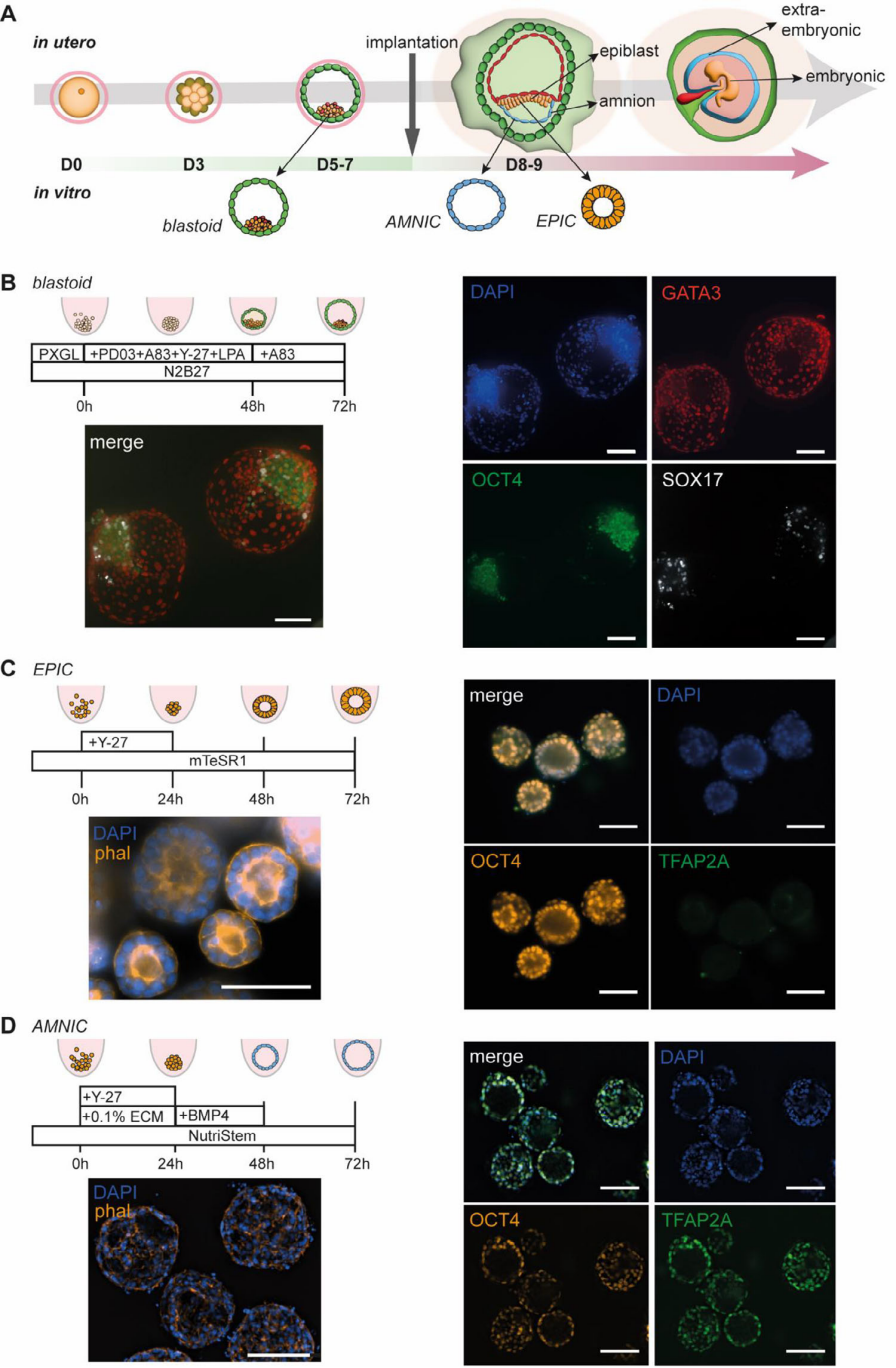
Epiblast, amnion and blastoid models. (A) Schematic of early human embryogenesis with the stem cell‐based models linked to the stages they represent, D indicates days post fertilization. (B) Schematic of the blastoid culture protocol used in this study, along with fluorescence microscopy images indicating all three blastocyst lineages through (immuno)staining (OCT4 indicating epiblast, SOX17 hypoblast and GATA3 marking trophectoderm). (C) Schematic of the culture protocol for EPICs, with one image of a DNA (DAPI) and F‐actin (phal) staining to illustrate the columnar cell morphology and one set of stainings indicating epiblast (OCT4) and amnion (TFAP2A). (D) Schematic of the culture protocol for AMNICs, with one image of a DNA (DAPI) and F‐actin (phal) staining to illustrate the squamous cell morphology and one set of staining indicating epiblast (OCT4) and amnion (TFAP2A). Scalebars depict 100 µm.

**FIGURE 2:**
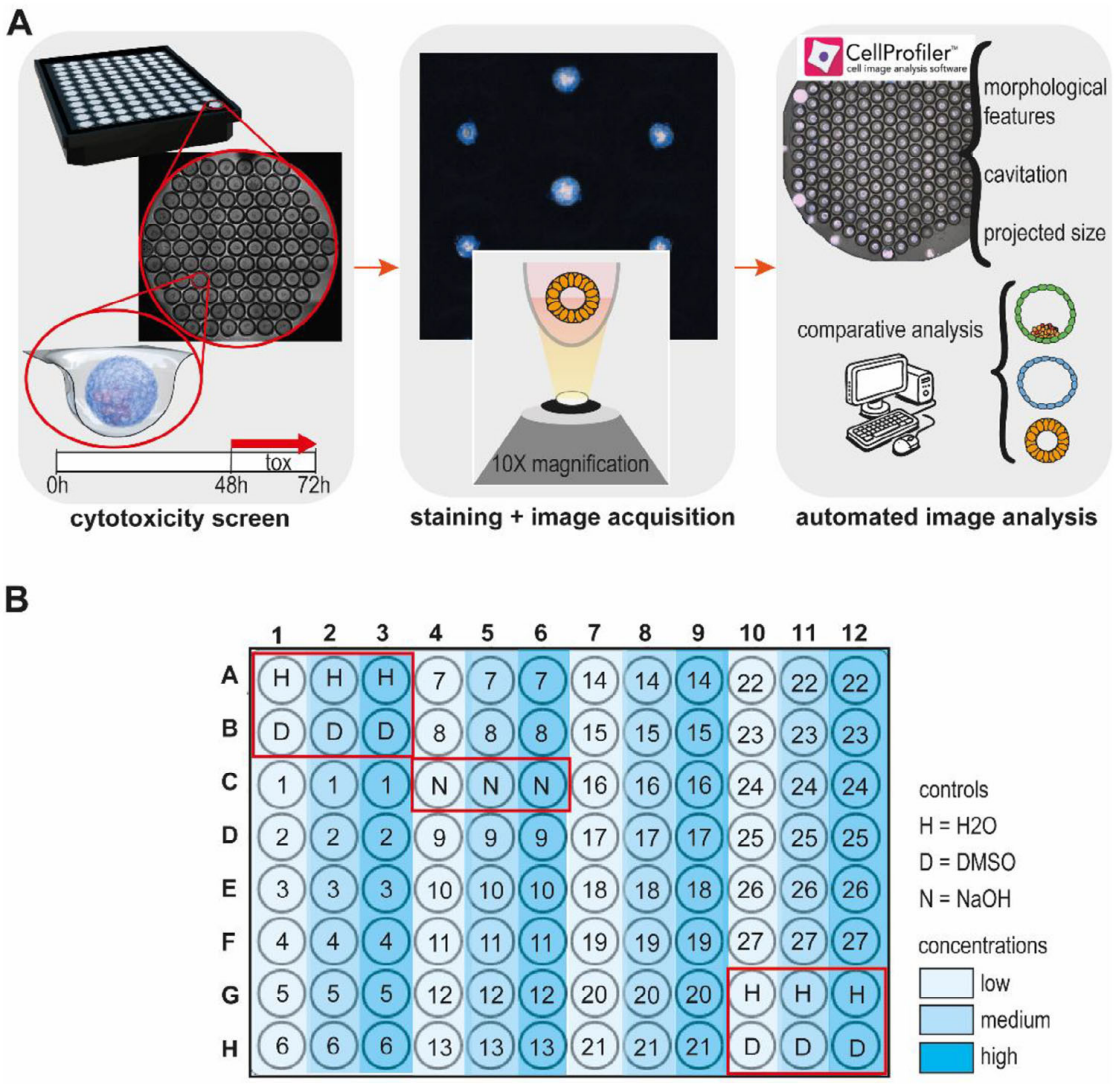
Experimental design of toxicity screening in thermoformed microwell arrays. (A) Outline of the three main stages of the screening, starting with the cell culture and exposure to the compound library in the microwells, followed by staining of DNA and F‐actin and image acquisition and ending with image analysis through the use of automated image analysis pipelines created in CellProfiler and subsequent comparative analysis of the results across the three models. (B) The lay‐out of the 96‐well plates with 169 microwells per well. Numbers indicate the compounds, H_2_O, DMSO, and NaOH indicate the control conditions and the blue marking indicates the three concentrations (low, medium, and high) per compound.

To start, we adapted established culture protocols of blastoids, epiblast, and amnion stem cell models, which recapitulate pre and early postimplantation stages of human development, respectively (Figure 1A), to thermoformed microwell platforms [30]. We formed blastoids by first expanding preimplantation stage (“naïve”) iPSCs using PXGL conditions [32] followed by 3D aggregation in the presence of a PAY (PD0325901, A‐83‐01, and Y‐27632), as previously described by Yanagida et al., but with the addition of Lysophosphatidic acid (LPA) as proposed by Kagawa et al. [18, 19]. Within 72 h of culture within microwells, the aggregates self‐organized into structures that morphologically closely resemble the blastocyst, with a cluster containing OCT4+ cells resembling the epiblast and SOX17+ cells indicative of the hypoblast, surrounded by a single layered cyst of GATA3+ trophectoderm‐like cells (Figure 1B). To mimic the epiblast in an early postimplantation stage, we used the H9 ESC line. We aggregated 20 cells per microwell in commercial mTeSR1 medium, which is typically used for the 2D expansion of hPSCs resembling an early postimplantation epiblast state. As reported by many others, we observed that these cells formed OCT4+ aggregates and underwent lumenogenesis in our suspension culture, reminiscent of the epiblast lumenogenesis reported in the early postimplantation stage [1, 25, 33]. Hence, we here refer to these aggregates with lumen as EPICs (Figure 1C). We modeled the amnion cell type in 3D by inducing the amniotic lineage in REUS2 ESC aggregates that were seeded with 50 cells per microwell and in the presence of 0.1% Geltrex. Amniogenesis was induced through exposure to BMP4 after 24 h, when the cells had aggregated. Following aggregation and lumenogenesis, the cells had a squamous epithelial morphology, rather than the columnar morphology of epiblast cells, after 72 h of culture. We confirmed using immunofluorescence staining that BMP4 treatment effectively upregulated TFAP2A (Figure 1D and Figure S1), a marker of the amniotic lineage, compared to the non‐BMP4‐treated EPICs (Figure 1C and Figure S1). We here refer to these amnion‐like embryoids as AMNICs (Figure 1D). By culturing structures in our thermoformed microwell arrays, we were able to generate up to 169 embryo‐like structures —blastoids, AMNICs, or EPICs — per well and thus 16224 structures per 96‐microtiter plate. This strategy (Figure 2A) ensures sufficient numbers for robust averaged measurements for subsequent toxicological screening.

### 2.2 Toxicological Screen Design and Image Analysis

For the toxicological screen, we selected 27 compounds to test at three different concentrations (Table 1). Concentrations were chosen per compound, based on previous literature describing toxicological tests performed on induced pluripotent stem cells, gastruloids, or mouse pluripotent stem cells [34, 35, 36]. Some of the compounds were reported to have teratogenic effects, some were shown to have no significant effect on embryonic development, and a few of the compounds have to our knowledge not been tested for developmental toxicity before (Table 1). We chose to include both teratogen (e.g., 5‐fluorouracil, busulfan, and thalidomide) and non‐teratogen (e.g., ascorbic acid, caffeine, glycolic acid) compounds to ensure our screening pipeline would be able to accurately assess every type of outcome, from well‐formed, intact structures to deformed or disintegrated structures. We also included several compounds hitherto untested on human iPSCs or embryo models, namely benztropine mesylate, methoxyacetic acid, and saquinavir mesylate. These were selected based on results from a mouse ESC‐based embryoid body screen. Most compounds were dissolved in DMSO or water, except for folic acid which was dissolved in 1 M NaOH. All three vehicles (DMSO, water, and NaOH) were therefore included as controls. The range of control concentrations was determined by the highest and lowest end concentrations present in the screen, as exact concentrations varied from compound to compound based on their stock concentration and the range of concentrations chosen per compound. For analysis, we compared every condition to the same or closest more concentrated vehicle control, ensuring a stringent comparison. In most cases, compounds could be compared to the lowest concentration vehicle control, even at the compounds’ highest concentrations. Additionally, we included our water and DMSO controls in two positions of the 96‐microtiter plate to be able to account for plate position effects (Figure 2B).

**TABLE 1:** An overview of the compound library. For each compound, the name, biochemical function, therapeutic application, toxicity classification, and the three tested concentrations in this screen are listed.

pos	Compound	Chemical property	Therapeutic use	Previously assessed as toxic	Vehicle	[High]	[Medium]	[Low]
20	Ascorbic acid	Vitamin C	To treat or prevent vitamin C deficiency	No	H_2_O	1.14 mM	284 µM	50 µM
16	Aspirin	Salicylate, COX inhibitor	Pain relief, anti‐inflammatory, fever reduction, blood thinner	No	DMSO	5 mM	500 µM	50 µM
5	Caffeine	Methylxanthine, adenosine receptor antagonist, phosphodiesterase inhibitor	Central nervous system stimulant, anti‐inflammatory	No	H_2_O	22.66 µM	18 µM	8 µM
21	Diphenhydramine	Ethanolamine‐based Histamine‐1 antihistamine	To treat allergies, insomnia, nausea, and cold symptoms	No	DMSO	784 µM	7.84 µM	784 nM
15	Folic Acid	Vitamin B9	To treat or prevent folate deficiencies and megaloblastic anemia	No	NaOH	2.27 mM	226.55 µM	22.66 µM
8	Glycolic acid	Alpha‐hydroxy acid (AHA)	Used in skincare as an exfoliant; to treat hyperpigmentation	No	H_2_O	5 mM	2.5 mM	1 mM
19	Isoniazid	Synthetic derivative of nicotinic acid	To treat tuberculosis	No	H_2_O	7.29 mM	729.19 µM	72.92 µM
18	Thiamine	Vitamin B1	To treat or prevent vitamin B1 deficiency	No	H_2_O	37.69 mM	3.77 mM	377 µM
14	Benztropine mesylate	Anticholinergic, antihistaminergic	To treat Parkinson's disease and extrapyramidal symptoms	?	H_2_O	200 µM	20 µM	2 µM
9	Methoxyacetic acid	Metabolite of ethylene glycol monomethyl ether (EGME)	Not therapeutically used; toxicant studied for reproductive effects	?	H_2_O	5 mM	1 mM	0.5 mM
25	Saquinavir mesylate	Protease inhibitor	Antiretroviral therapy for HIV/AIDS	?	DMSO	10 µM	1 µM	100 nM
26	2,4,6‐triiodophenol (TIP)	Iodinated phenolic compound	Antimicrobial, potential uncoupler of oxidative phosphorylation	Yes	DMSO	100 µM	50 µM	10 µM
27	5‐Fluorouracil	Nucleoside metabolic inhibitor	Chemotherapy	Yes	H_2_O	500 µM	100 µM	50 µM
7	Acetazolamide	Carbonic anhydrase inhibitor	To treat glaucoma, altitude sickness, and epilepsy	Yes/?	DMSO	121 µM	100 µM	67 µM
1	Bosentan	Endothelin receptor antagonist	Antihypertensive, to treat pulmonary artery hypertension	Yes	DMSO	40 µM	18 µM	4 µM
24	Busulfan	Alkylating agent	Chemotherapy for leukemia	Yes	DMSO	40.6 µM	4.06 µM	406 nM
17	Carbamazepine	Tricyclic compound chemically related to tricyclic antidepressants (TCA)	Anticonvulsant and analgesic	Yes	DMSO	423.25 µM	42.33 µM	423.25 nM
23	Cytosine arabinoside	Pyrimidine nucleoside analog	To treat leukemia and lymphoma	Yes	DMSO	5 µM	500 nM	50 nM
22	Dexamethasone	Corticosteroid, glucocorticoid	Anti‐inflammatory, immunosuppressant, to treat allergies and shock	Yes	DMSO	1 µM	100 nM	10 nM
4	Hydroxyurea	Ribonucleotide reductase inhibitor	To treat sickle cell anemia and cancer	Yes	DMSO	100 µM	67 µM	33 µM
3	Ibuprofen	Nonsteroidal anti‐inflammatory drug (NSAID), COX inhibitor	Analgesic, anti‐inflammatory, antipyretic	Yes	DMSO	250 µM	120 µM	63 µM
2	Phenytoin	Hydantoin derivative	Anticonvulsant	Yes	DMSO	20 µM	5 µM	1 µM
6	Phthalic acid	Aromatic dicarboxylic acid	Not therapeutically used; industrial use in plastics	Yes	DMSO	100 µM	50 µM	1 µM
13	Propafenone hydrochloride	Class IC antiarrhythmic drug	To treat ventricular and supraventricular arrhythmias	Yes	DMSO	30 µM	1 µM	500 nM
10	Thalidomide	Immunomodulatory and antiangiogenic	To treat multiple myeloma and erythema nodosum leprosum	Yes	DMSO	100 µM	10 µM	0.1 µM
12	trans Retinoic Acid	Vitamin A derivative (all‐trans retinoic acid, ATRA)	To treat acne and acute promyelocytic leukemia (APL)	Yes	DMSO	100 nM	33 nM	0.4 nM
11	Valproic Acid (VPA)	Histone deacetylase inhibitor, GABA modulator	Anticonvulsant and mood stabilizer; to treat epilepsy and bipolar disorder	Yes	DMSO	1 mM	333 µM	4 µM

Blastoids, AMNICs, and EPICs were cultured for a total of 72 h, with exposure to the compounds at three concentrations for a 24 h‐window from 48 h post‐seeding. For the in‐situ imaging‐based analysis of toxicity of each condition, cells in the plates were fixed at 72 h and stained for their nuclei (Hoechst) and F‐actin (phalloidin‐568; Figure 2A). The image acquisition was automated using Nikon's JOBS software using autofocusing per image. One montage image per well of the 96‐microtiter plate was generated in three channels (brightfield, Hoechst, and phalloidin) for the EPICs and AMNICs. Given the size of the blastoids, a Z‐stack of seven planes was generated, rather than a single focused image. These Z‐stacks were consolidated into a single focused image using Nikon's GA3 post‐acquisition batch processing module, ensuring that both the blastoids’ outer layers and their inner cell mass‐like structures were in focus.

Next, a CellProfiler image analysis pipelines were developed for each individual embryo model. In all cases, the first part of the pipeline was designed to identify all Hoechst+ objects. For these objects, area, shape, and intensity features in the three channels were measured. Then for the EPICs and AMNICs, the CellProfiler Analyst software was used to train a supervised machine‐learning algorithm to distinguish intact structures (example in Figure S2A) from structures with a disrupted morphology based on measured features (examples in Figure S2B and C). From these training sessions, rules were derived to filter out the intact structures from the total group of Hoechst+ objects. Once fed in into CellProfiler, these rules were used to quantify the yield and extract the average projected area of well‐formed EPICs and AMNICs per image. For blastoids, instead of relying on machine learning, we developed an image‐based pipeline through an informed and rationale‐driven selection of features, with manual assessment of potential filtering criteria within the CellProfiler software. Yield measurements were validated with manual scoring in one plate for all three embryo models(Figures S3–S5). Correlation analysis indicated for a strong correlation between manual and automatic yield measurements for all three embryo model pipelines (Pearson correlation coefficients ranged between 0.8744 and 0.8849; Figures S3C, S4C, and S5C)

For a fair comparison, all yield and area outputs were normalized to the vehicle controls with concentrations similar or higher than the screening condition (Table S1). Statistical testing was performed on the normalized values. All in all, we were able to create image analysis pipelines for blastoids, AMNICs, and EPICs, enabling us to analyze screening data sets of 27 compounds at three concentrations for each of these models. Importantly, through normalization we were able to compare results across the three complementary embryo models.

### 2.3 Blastoid Toxicity Screen

**FIGURE 3:**
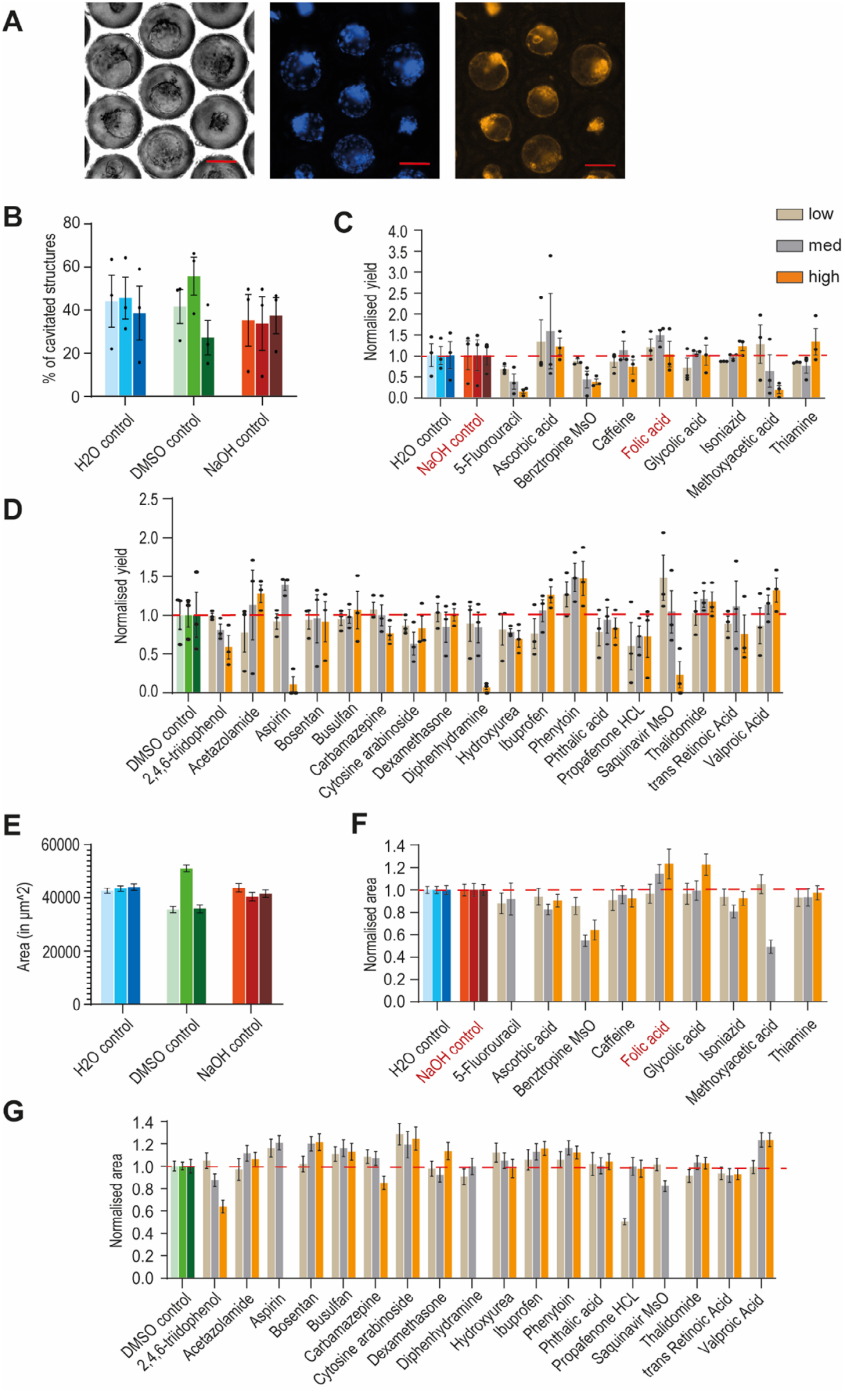
Morphotoxicity screen on blastoids. (A) Example images of control blastoids in all three channels (brightfield, Hoechst, phalloidin‐568), scale bar 200 µm. (B) Yield of cavitated structures in vehicle control conditions, with concentrations from low to high for each vehicle, from left to right as indicated with darkening of the color. (C) Normalized blastoid yields in conditions with H_2_O or NaOH‐dissolved compounds. (D) Normalized blastoid yields in conditions with DMSO‐dissolved compounds, E) Area of blastoids measured in µm^2 in vehicle control conditions, sample size dependent on the number of blastoids per condition. (F) Normalized blastoid area in conditions with H_2_O or NaOH‐dissolved compounds and (G) Normalized blastoid area in conditions with DMSO‐dissolved compounds. *n* = 3.

Blastoids were screened using microwell plates with the toxic compound layout (Figure 2B). The features selected to define intact blastoids were shape (e.g., solidity) and Hoechst and phalloidin intensity features (Figure 3A and Data S1). Through trial‐and‐error adjustments to the thresholding values in the filtering step, we ensured a stringent selection of undisrupted blastoids that correlated with our manual measurements (Figure S3A–C).

When we checked the vehicle controls, we noticed yields were variable between replicates and relatively low (averaging between 34% and 56%). We confirmed that both yields and projected areas in vehicle control conditions were not significantly different from regular medium control (Figure S3D, E). Therefore, this variability can be attributed to inherent culture heterogeneity, which the blastoid model is prone to. Serendipitously however, the relatively low yields in this screen increased the dynamic range at the upside to detect potential beneficial effects toward blastoid formation. For instance, medium DMSO controls showed a considerable higher yield of up to 66.2% compared to other control conditions (Figure 3B), which is in line with the work of Alsolami et al. that describes the use of DMSO to increase blastoid yields [37]. Also, ascorbic acid (53.9% on average at the high concentration) and to a lesser extent thiamine (43.4% on average at the high concentration) showed higher rates of well‐formed blastoids compared to their controls (Figure 3C). Interestingly, while in some compounds (5‐fluorouracil, benztropine mesylate, and 2,4,6‐triidophenol) yields only decreased with increasing concentrations, there were also several examples of increased yield at low concentrations, but decreased yields at medium and high concentrations, such as methoxyacetic acid and saquinavir mesylate.

Another striking case was VPA, which strongly increased the blastoid yield (Figure 3D; average yield at high concentration: 56.9%). High concentrations of ibuprofen and phenytoin increased blastoid yields as well (average yields of 50.6% and 57.9% at their highest concentrations, respectively), despite having been reported toxic when tested on gastruloids, which mimic the postimplantation epiblast as it undergoes gastrulation (Table 1) [34]. On the other hand, aspirin and diphenhydramine, two compounds that are not generally considered toxic at the tested concentrations [35, 38, 39], clearly disrupted blastoid formation at their highest concentrations (average yields of 4.3% and 2.5%, respectively).

To determine whether compounds affect blastoid size, we measured the average projected area of blastoids. For robust analysis, we removed measurements of conditions with a normalized yield lower than 0.2. The vehicle controls showed consistent projected areas, despite the variability of the yields (Figure 3E). Among the water‐dissolved compounds benztropine mesylate and glycolic acid at their low concentrations (1 mM and 2 µM, respectively) significantly decreased the blastoids’ projected area (Tamhane's T2 post hoc test, *p* = 0.0068 and *p* <0.0001, respectively; Figure 3F and Table S2). Thiamine similarly reduced the blastoid projected area at its medium concentration (3.77 mM, Tamhane's T2 post hoc test, *p* = 0.0135). Although ibuprofen, phenytoin and VPA show trends of increasing blastoids’ projected area with increasing concentrations, none of these conditions were significantly different from controls (Figure 3G and Table S2). Only the high concentrations of 2,4,6‐triiphenol and carbamazepine (100 and 423.25 µM, respectively) and the low concentration of propafenone hydrochloride (500 nM) significantly impacted the projected area, leading to on average smaller areas (Tamhane's T2 post hoc test, *p* <0.0001, *p* = 0.0003 and *p* <0.0001, respectively; Figure 3G and Table S2).

### 2.4 AMNICs Toxicity Screen

**FIGURE 4:**
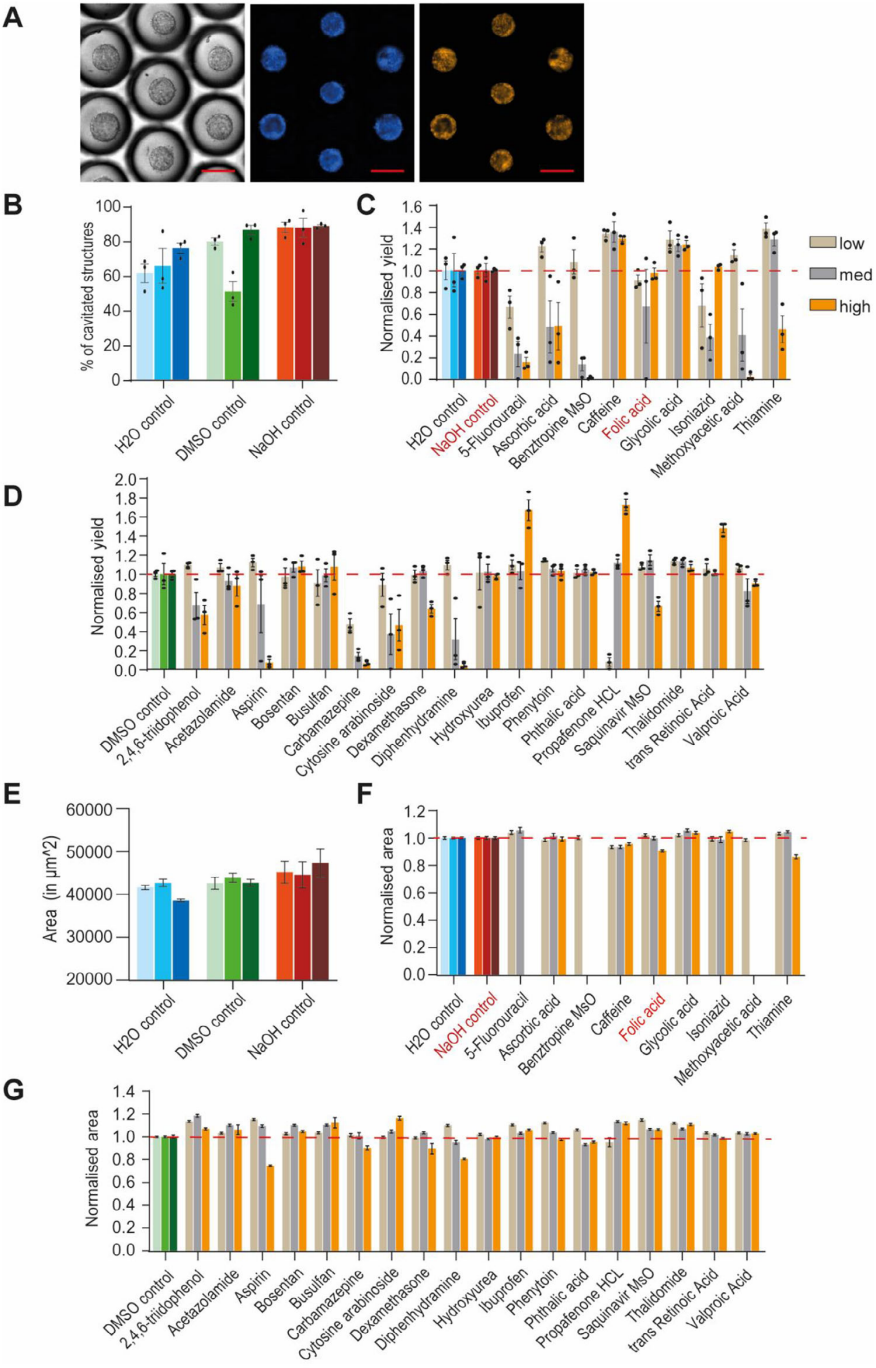
Morphotoxicity screen on AMNICs. (A) Example images of control AMNICs in all three channels (brightfield, Hoechst, and phalloidin‐568), scale bar 200 µm., (B) Yield of cavitated structures in vehicle control conditions, with concentrations from low to high for each vehicle, from left to right as indicated with darkening of the color. (C) Normalized AMNIC yields in conditions with H_2_O or NaOH‐dissolved compounds. (D) Normalized AMNIC yields in conditions with DMSO‐dissolved compounds (E) Area of AMNICs measured in µm^2 in vehicle control conditions, sample size dependent on the number of AMNICs per condition (F) Normalized AMNICs area in conditions with H_2_O or NaOH‐dissolved compounds and (G) Normalized AMNIC area in conditions with DMSO‐dissolved compounds. *n* = 3.

Next, we performed the same screen on AMNICs cultured in thermoformed microwells (Figure 4A). In the AMNICs pipeline, the CellProfiler Analyst (CPA) machine‐learning algorithm filtered intact from disrupted AMNICs based on texture and intensity measurements in the Hoechst channel (Contrast and StdIntensityEdge) as well as radial distribution patterns in the F‐actin channel (ZernikeMagnitude) and shape descriptors (HuMoment and ZernikeMoment; Data S2 and S3). The pipeline was validated by comparing manual and automated scoring of one of the three replicate plates (Figure S4A–C).

While AMNICs formed well in water and NaOH controls (yields between 61.8%–76.3% for water, 88.1%–89.1% for NaOH), as well as the low and high concentration DMSO controls (yields of 80.1% and 86.9%, respectively). Medium DMSO vehicle control however resulted in a notably reduced yield (overall average of 51.3%), though again this was not reflected in our vehicle control versus medium experiment (Figure 4B and Figure S4D). In terms of yield, water and NaOH dissolved conditions that significantly impacted the AMNICs’ formation rate were the medium and high concentrations of 5‐fluorouracil, benztropine mesylate and methoxyacetic acid (Dunnett's post hoc test, *p* <0.02). On the other hand, though not significantly, caffeine (at all concentrations tested) and glycolic acid (at medium and high concentrations) seemed to improve yields (yields of ≥76%; Figure 4C). Among the DMSO‐dissolved compounds, 2,4,6‐triidophenol (TIP), aspirin, carbamazepine, cytosine arabinoside and diphenhydramine significantly reduced AMNIC yields. TIP and aspirin reduced yields only at their highest tested concentrations (Dunnett's post hoc test, *p* = 0.0355 and *p* <0.0001, respectively). Carbamazepine reduced yields at all tested concentrations (Dunnett's post hoc test, *p* = 0.0031 at low and *p* <0.0001 at medium and high concentrations), while cytosine arabinoside and diphenhydramine decreased yields at their medium and high concentrations (Dunnett's post hoc test, *p* = 0.0002 and *p* = 0.0024 for cytosine arabinoside and *p* <0.0001 for both medium and high concentrations of diphenhydramine). Interestingly, propafenone hydrochloride reduced yields to nearly zero at its low concentration (average yield of 5.1%), while increasing the yield to 88.6% at its high concentration (Figure 4D).

We observed variability in AMNIC projected area across vehicle controls, with larger average areas in the NaOH vehicle compared with H_2_O or DMSO, and differences between the high and the low and medium H_2_O vehicle conditions (Figure 4E and Figure S4E). Because the vehicle controls were not iso‐osmotic (and the NaOH vehicle may also affect pH), part of this variability may reflect vehicle‐related osmotic/pH effects. Therefore, compound effects are primarily evaluated relative to the matched vehicle control used for the respective stock solution. Similar to the approach for the blastoid measurements, we removed projected area averages of conditions with yields lower than 0.2, due to their small sample size. All tested concentrations of caffeine significantly reduced the projected area of AMNICs (Tamhane's T2 post hoc test, *p*≤0.01). Exposure to the high thiamine concentration also significantly decreased projected area size (Tamhane's T2 post hoc test,*p* <0.0001). Notably, all other conditions that significantly differed from the water control resulted in slightly larger AMNICs, rather than smaller. These conditions were medium glycolic acid (2.5 mM), high isoniazid (7.29 mM), and medium thiamine (3.77 mM; Tamhane's T2 test, all *p*≤0.0133; Figure 4F and Table S2).

Among the DMSO dissolved compounds, we observed that there were several conditions in which AMNIC projected area was decreased. However, most of these were based on small sample sizes, as AMNIC formation yields in these conditions were low, and thus not considered significant. Only phthalic acid at its medium and high concentrations significantly decreased AMNIC projected area (Tamhane's T2 post hoc test, *p* <0.0001 and *p* = 0.0012, respectively). The majority of significantly altered conditions (low diphenhydramine and phthalic acid, low and medium aspirin and phenytoin, low and high ibuprofen, medium acetazolamide and busulfan, medium, and high bosentan and propafenone hydrochloride, high concentration of cytosine arabinoside and all concentrations of TIP, saquinavir mesylate, and thalidomide) had increased AMNICs’ projected areas compared to their control (Tamhane's T2 post hoc test, *p*≤0.017, Figure 4G and Table S2). These increased AMNICs’ projected areas did not correlate with a significant increase or decrease in AMNIC yield.

### 2.5 EPICs Toxicity Screen

**FIGURE 5:**
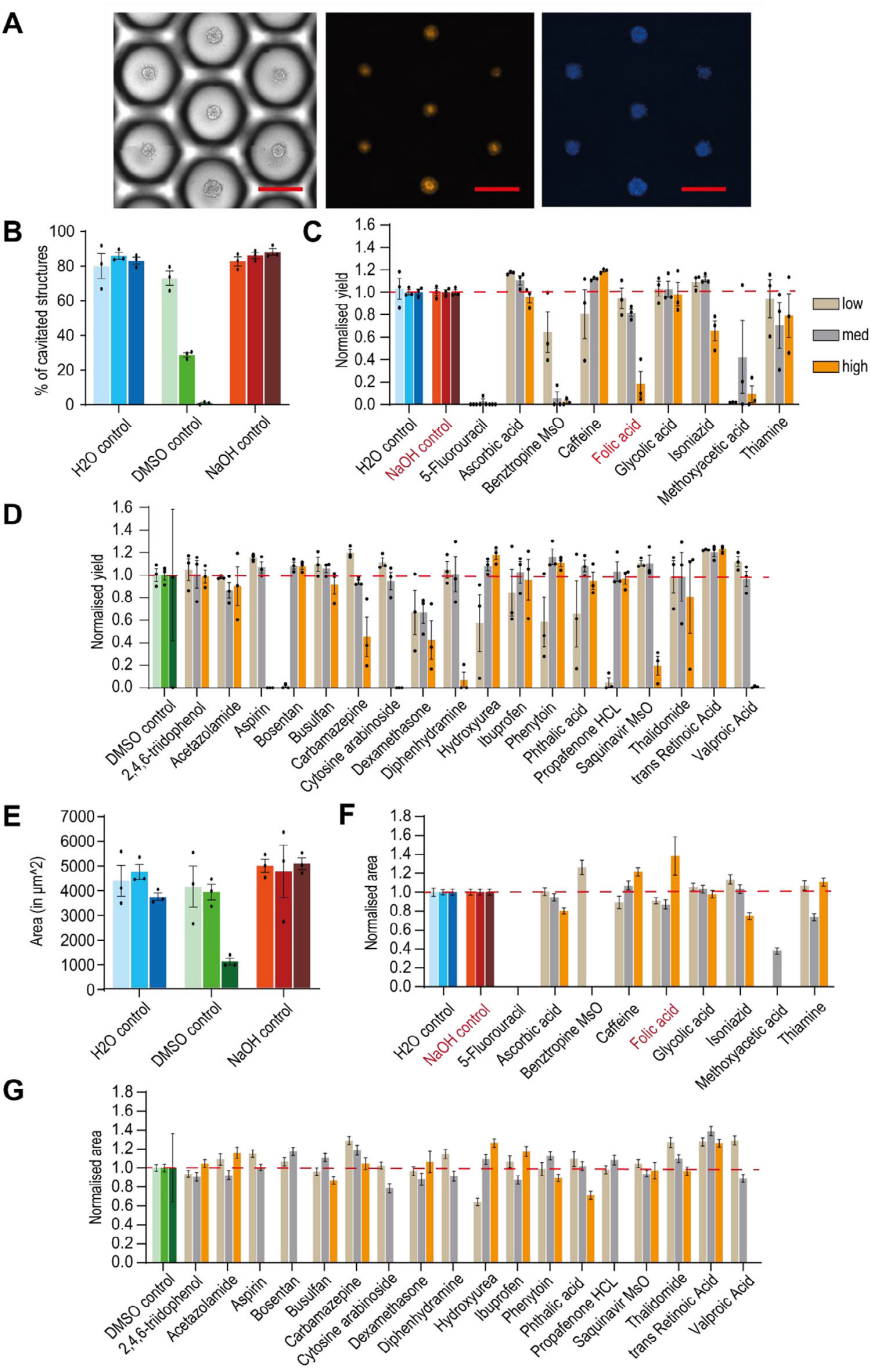
Morphotoxicity screen on EPICs. (A) Example images of control EPICs in all three channels (brightfield, Hoechst, and phalloidin‐568), scale bar 200 µm. (B) Yield of cavitated structures in vehicle control conditions, with concentrations from low to high for each vehicle, from left to right as indicated with darkening of the color. (C) Normalized EPIC yields in conditions with H_2_O or NaOH‐dissolved compounds. (D) Normalized EPIC yields in conditions with DMSO‐dissolved compounds. (E) Area of EPICs measured in µm^2 in vehicle control conditions, sample size dependent on the number of EPICs per condition. (F) Normalized EPICs area in conditions with H_2_O or NaOH‐dissolved compounds and (G) Normalized EPIC area in conditions with DMSO‐dissolved compounds. *n* = 3.

A third screen was performed on EPICs. The main characteristics of EPICs are the densely packed cells within these embryoids and the inner lining of the lumens clearly marked with F‐actin (Figure 5A). Hence, the CPA algorithm identified well‐formed EPICs using intensity features, including DAPI's radial distribution (MeanFrac and RadialCV) and intensity of DAPI and phalloidin on the edge of detected structures (StdIntensityEdge and MinIntensityEdge; Data S4 and S5). First, we checked the control conditions, and we noticed the water and NaOH controls formed EPICs consistently, regardless of their concentration (water control yields between 77.6%–86.0% and NaOH control yields between 82.7%–88.2%). The only exception was the low concentration water control in the top corner of the plates, which varied between plates and was always low compared to other water controls. Although the low concentration DMSO control matched water and NaOH controls (average yield of 73.0%), medium and high DMSO controls consistently disrupted EPICs formation (average yields of 28.8% and 0.8%, respectively; Figure 5B). This disruption was not significant when we compared vehicle controls to the medium control (Figure S5D).

In the water‐dissolved compound group, both 5‐fluorouracil and methoxyacetic acid significantly decreased EPICs yields at low, medium and high concentrations (Dunnett's post hoc test, *p*≤0.0035). Besides these two compounds, benztropine mesylate lowered EPICs’ yields at medium and high concentrations (Dunnett's post hoc test, both *p* <0.0001), while folic acid only blocked EPICs’ formation at its highest tested concentration (Dunnett's post hoc test, *p* <0.0001; Figure 5C). Exposure to the DMSO‐dissolved compounds aspirin, bosentan, cytosine arabinoside, diphenhydramine, propafenone hydrochloride, saquinavir mesylate, and VPA was toxic to EPICs at high concentrations (Dunnett's post hoc test, *p*≤0.0028; Figure 5D).

EPICs had more or less the same projected area across controls, with exception of the high DMSO control, where the average area was much smaller (956,1 µm^2^ compared to 4164,5 and 3972,3 µm^2^ at low and medium concentration; Figure 5E). Notably, we found all vehicle conditions (with the exception of medium concentration DMSO) result in EPICs significantly smaller than EPICs formed in medium control (Figure S5E). Among the water dissolved compounds, there were no measurements of 5‐fluorouracil or the high concentration of benztropine mesylate, as no EPICs remained in these conditions following exposure. Measurements of conditions with normalized yields below 0.2 were removed like they were in the blastoid and AMNIC screen. Several compounds significantly decreased the projected area in EPICs, namely ascorbic acid and isoniazid at their high concentrations (Tamhane's T2 post hoc test, both *p* <0.0001) and thiamine at its medium and high concentrations (Tamhane's T2 post hoc test, *p* <0.0001 and *p* = 0.0081, respectively). On the other hand, the medium concentration of folic acid (226.55 mM), as well as the low concentration of benztropine mesylate (2 µM) increased EPICs’ projected area (Tamhane's T2 post hoc test, *p* = 0.0004 and *p* <0.0001, respectively; Figure 5F). From the DMSO‐dissolved compounds, exposure to diphenhydramine and hydroxyurea at their low concentrations (784 nM and 33 µM; Tamhane's post hoc test, *p* = 0.0002 and *p* <0.0001, respectively) resulted in EPICs that were significantly smaller than controls (Figure 5G and Table S2). Other statistically significant hits were low concentrations of aspirin, carbamazepine, saquinavir mesylate, thalidomide and VPA, medium concentrations of carbamazepine, phenytoin and hydroxyurea, as well as the high concentrations of hydroxyurea and ibuprofen, and all tested concentrations (low, medium, and high) of trans retinoic acid (Tamhane's T2 post hoc test, *p*≤0.017). In all the aforementioned conditions, EPICs showed an increase in projected area. Overall, comparison between yields and projected area suggests a positive correlation. However, several exceptions were observed, such as folic acid, which at its highest concentration, decreased the yield while the average EPIC projected area increased, albeit not significantly. Similarly, dexamethasone and saquinavir mesylate lead to yield reductions —yet not all statistically significant—while EPIC projected area stayed roughly the same. All in all, these findings suggest that different mechanisms, depending on the compound, are at play.

### 2.6 Comparisons Between Embryo Models

**FIGURE 6:**
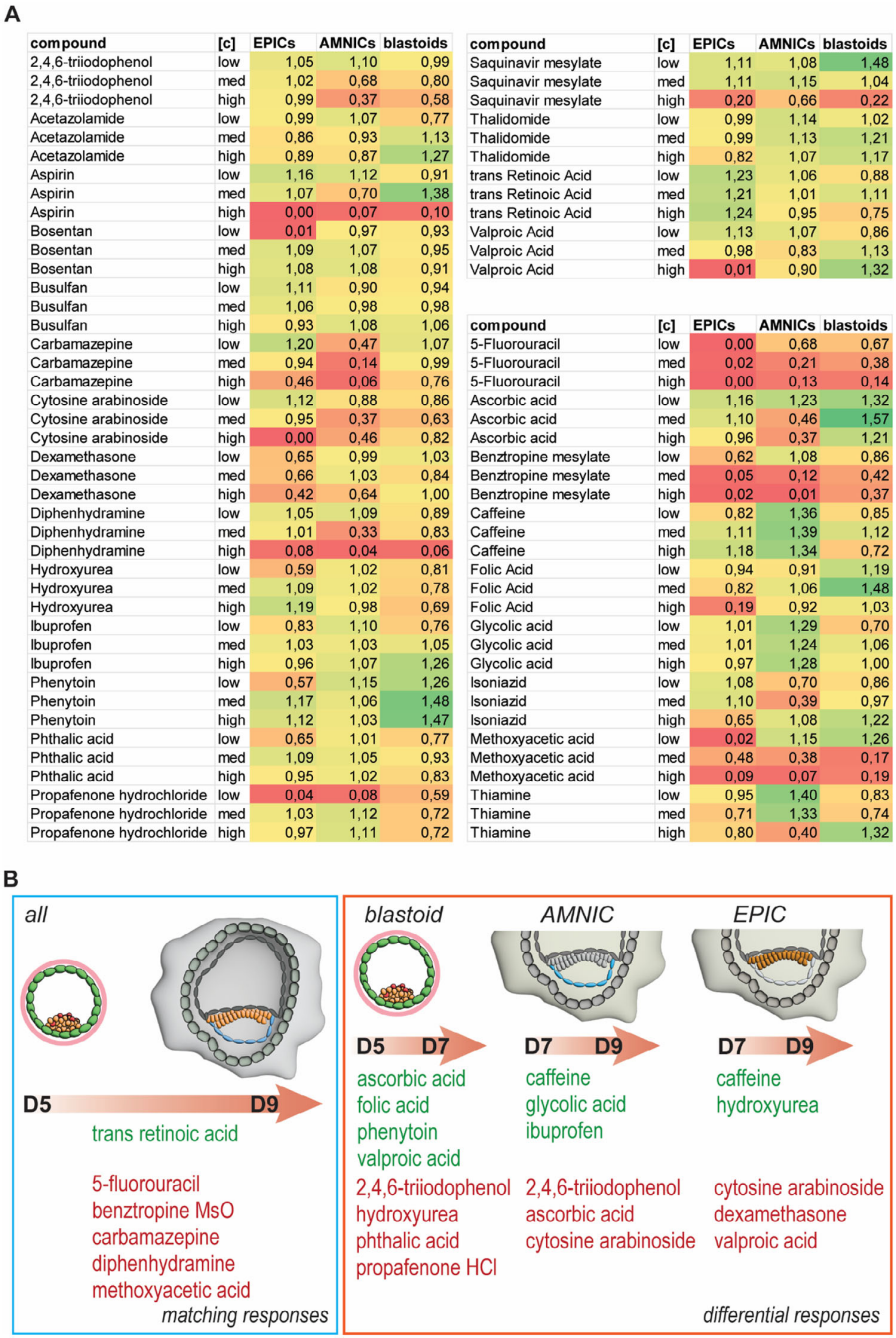
A summary of average normalized yields across blastoids, AMNICs, and EPICs. (A) Heatmap of all normalized yields across the three models. Green indicates a relative increase in yield compared to control conditions, while red indicates a decrease in yield. (B) Schematic indicating the time range and/or cell type affected by the tested compounds. The box outlined in blue shows the developmental timeline that is covered by these embryo models together and compounds that affected the yields of all models similarly are listed here (matching responses). Effects of compounds on yield that varied between the three models are listed in the orange box (differential responses). In this schematic, compounds listed in green increased yields while compounds in red decreased yields.

One of the main strengths of performing the same developmental toxicity screen on a complementary set of embryo models spanning different stages and cell lineages is that stage and lineage‐specific sensitivities to compounds can be identified. By comparing the preimplantation blastoids with postimplantation EPICs and AMNICs, we observed that a number of compounds, namely 5‐fluorouracil, benztropine mesylate, carbamazepine, diphenhydramine, and methoxyacetic acid, reduced yields in all three embryo models. On the other hand, phenytoin and trans retinoic acid were examples of compounds that overall increased the yields of all three embryo models (Figure 6).

Beside these similar responses, notable differences emerged between blastoids, EPICs, and AMNICs in their response to compounds. An example is ascorbic acid, which consistently enhanced blastoid yields across all concentrations, whereas EPICs were barely affected and AMNICs showed a decrease in yields with increasing concentrations of this compound. Further differential effects were observed for VPA and thiamine, that had a positive effect on blastoid yields (with average yields increasing from low to high concentration, from 38.8% to 56.9% for VPA and from 36.8% to 43.4% for thiamine), yet had a limited or strongly detrimental effect on the yields of AMNICs (VPA yields decreased from 85.8% to 72.3% and thiamine yields decreased from 85.2% to 31.3%) and EPICs (low to high concentrations of VPA yields decreasing from 81.9% to 0.07% and thiamine yields from 73.0% to 65.9%). Moreover, we also observed cases where one of the postimplantation models reacted more similar to the blastoids than to the other postimplantation model. TIP reduced blastoid yields (from 41.4% to 21.9%) and AMNIC yields (from 88.0% to 29.2%) but did not affect the yield of EPICs (average yields between 72.1% and 76.5%). In the case of ibuprofen, blastoid and AMNIC yields were improved compared to control at the high concentration (50.6% vs. 41.6% DMSO control for blastoids, 85.7% vs. 51.3% for AMNICs) while EPIC yields were unaffected (70.0% vs. 73.0%).

Notably, several compounds elicited distinct responses across the three models. Propafenone hydrochloride for example, reduced EPIC and AMNIC yields at its low concentration (3.5% and 6.1% yield respectively), but at medium and high concentrations barely affected EPICs (75.3% at medium and 70.9% at high vs. 73.0% and 28.8% in DMSO vehicle controls) and increased AMNIC yields (89.5% at medium and 88.6% at high vs. 75.3% and 51.3% in DMSO vehicle controls). Hydroxyurea increased EPIC yields with increasing concentrations (from low to high: 42.0%, 78.9% and 86.2%), slightly reduced blastoid yields at all concentrations (from low to high: 36.8%, 32.8%, and 30.0% compared to 41.6% DMSO control), while AMNICs remained unaffected at every concentration (from low to high: 81.3%, 82.0%, and 78.5% compared to 80.1% DMSO control).

Although projected area, as proxy for size, varied across conditions, there was minimal overlap between the models. The blastoids projected area only significantly changed in a small group of conditions, while AMNICs’ projected area was affected by more than half of the compounds. No clear pattern or trend emerged as we evaluated the overlaps between models and compared yield and projected area changes across conditions. For example, both AMNICs’ and EPICs’ average projected area increased in response to saquinavir mesylate and thalidomide acid at a low dosage. For both AMNICs and EPICs, neither condition had a significantly altered yield. Taken together, these observations confirm across the embryo models that area and yield generally are not associated per se, which suggests the relationship varies between compounds depending on the underlying modes of action for each compound. To illustrate the variation in modes of action and subsequent effects, we performed a cleaved Caspase3 staining (Figure S6) on the high concentration conditions of benztropine mesylate, carbamazepine and folic acid for all three embryo models and saw that toxic effects ranged from embryo models that were fully Caspase3 positive and disintegrated to structures that were largely negative for Caspase3 and only lost their cavitated morphology.

All in all, through our comparisons between embryo models we could demonstrate how different lineages of early embryonic stages can respond differently to the same (toxic) condition, thus underscoring the need for testing across multiple embryo models for a comprehensive overview of a compound's developmental toxicity.

## 3 Discussion

**FIGURE 7:**
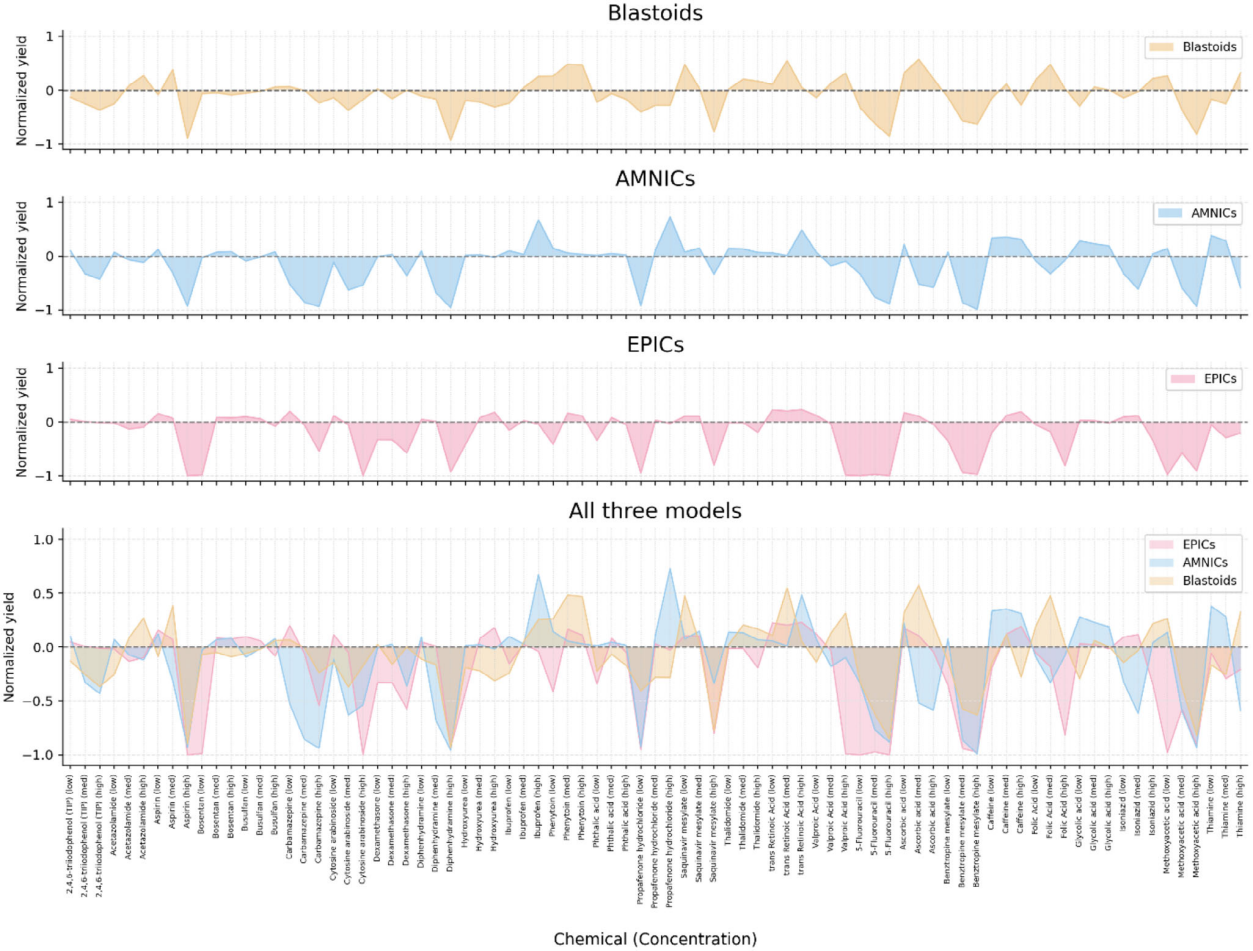
The concept of comparing embryo models visualized with normalized yields of embryo models following compound exposure. Yields of blastoids, AMNICs, and EPICs are depicted after linear modification to their respective controls. Blastoid yields are depicted in yellow, AMNICs are depicted in blue, and EPICs in pink. The fourth graph shows the overlapping graphs of the three models to illustrate similarities and differences between the different models.

This study introduces the concept of using multiple 3D human embryo models to evaluate morphotoxicity of compounds during an (early) developmental time window. Here, we focus specifically on screening blastoids, EPICs, and AMNICs to assess morphotoxicity in the peri and early postimplantation stage. By screening these models in parallel, we aim to address important gaps in assessing early developmental risks. Integrated with high‐throughput thermoformed microwell platforms and automated image analysis, this approach enables rapid, human‐relevant screening of pharmaceutical compounds and environmental toxicants. Through the comparison between models, it is possible to reveal lineage and stage‐specific toxic effects (Figure 7). This offers unique insights into developmental pathophysiology and pregnancy complications, paving the way for safer drug development and consumer product testing.

One of the most striking examples of stage and lineage‐specific effects found in this study was the detrimental effect of ascorbic acid on AMNIC yields, while it supported formation of EPICs and blastoids. Little is known about the role of ascorbic acid in early human development, but addition of ascorbic acid to IVF culture media for preimplantation embryos is correlated with improved blastocyst development in bovine, ovine and porcine context [40, 41, 42]. Similarly, VPA, a histone deacetylase inhibitor, appeared detrimental for the formation of AMNICs and EPIC representing early postimplantation morphogenesis, yet boosted blastoid yields. VPA exposure has been shown to lead to hyperacetylation [43]. While this may be favourable during preimplantation development [44], by facilitating epigenetic resetting, at later stages VPA disrupts developmental processes and has thus been classified teratogenic [45, 46]. Another example of stage‐specific effects is phenytoin. This anticonvulsant drug is linked to developmental defects in mice and associated with craniofacial and neurodevelopmental anomalies in humans [47, 48]. Surprisingly, phenytoin enhanced the formation of all three embryo models in this study. These three examples of ascorbic acid, VPA and phenytoin illustrate the necessity of testing a range of developmental stages and embryo models for comprehensive understanding of developmental toxicity. Understanding the human stage‐specific effects of teratogens like VPA and phenytoin is crucial for developing strategies to mitigate risks associated with prenatal exposure. Unexpectedly, findings from developmental toxicity screenings may even improve embryo model cultures through the identification of compounds that increase embryo model formation rates and reproducibility. Here we found several compounds, such as ascorbic acid, glycolic acid, retinoic acid, and thiamine, that enhanced the formation rate of either one or multiple of the three embryo models. These findings may contribute to the development of more robust embryo models, ultimately advancing the field of toxicology. As of yet, variability in embryo model formation—specifically in the case of blastoids—is still a hurdle for the use of embryo models in large‐scale, standardized applications, such as safety testing in drug development [49].

Furthermore, this study confirms the importance of species‐specific toxicity screening. In several cases, compounds that were reported to be teratogenic in mouse embryos (e.g., acetazolamide and busulfan) had little to no effect on the yields of blastoids, EPICs, and AMNICs, demonstrating a differential response between human embryo‐like cells and widely used rodent models of peri‐ to early post‐implantation stage [50, 51]. This again showcases the need for relevant 3D human embryo models to assess developmental toxicity.

Based on our results, we propose that future studies on developmental toxicity with embryo models should encompass multiple embryo models and comparative analysis between them to facilitate a comprehensive overview of a compound's risk to early embryogenesis. This approach could be adapted to other developmental stages, for example, day 7–14 of human embryo development for which many models have recently emerged [27, 52, 53, 54]. If the strategy of complementary embryo models for toxicity screening is to be widely adopted in the future, a new framework of guidelines will be required to help scientists decide on the number of models needed for their study and how to determine the suitability of any given model to be included in a study. This may eventually set a new standard for drug safety regulations.

While the methodology described in this study provides a solid proof‐of‐concept for initial high‐throughput screening, several elements could be further optimized to strengthen the robustness of the findings. In this work, we evaluated three cell lines with a limited number of replicates. Future studies could expand these assays to a broader panel of cell lines to better capture source variability and improve generalizability. Particularly testing of a broader range of compound concentrations across cell lines could help identify differences in responsiveness between cell lines. In addition, follow‐up validation assays would help confirm and further characterize the effects identified in the screening results. From a technical perspective, several features could be investigated to further improve our pipeline. First, variability in imaging focus across microwells in the current pipeline could be addressed through optimized autofocus steps or better filtering of images to ensure more reliable data collection. Second, the current image analysis relies on manual thresholding or manually trained machine learning algorithms. The inherent bias this causes could be removed by introducing adaptive or automated thresholding approaches.

Furthermore, readouts could be made more extensive through the extraction of a wider range of features from the CellProfiler image analysis or by using more refined approaches. These include more detailed phenotypic fingerprinting, improved identification of morphological “fluidity” to capture a broader spectrum of similar yet distinct morphologies, and deep‐learning‐based analysis for precise comparisons of morphological and lineage‐specific developmental responses to toxic compounds [55]. Similar image‐based readouts are currently rolled out in the field of assisted reproductive technologies (ART) [56]. In addition, mechanisms of action of morphotoxic compounds could potentially be inferred from the phenotypic changes when exposed to these compounds [14, 57]. Additional assays such as for cell viability, proliferation, metabolic activity and gene expression analysis, may further inform on the cytotoxic and morphotoxic modes of action that manifest in reduced formation rates and inhibited growth of the embryo models. Finally, beyond looking at the embryonic stages in isolation, the addition of maternal tissues to model maternal‐fetal interactions could integrate the kinetics of toxicant transfer across the placental barrier and metabolism into the response to toxicant exposure of embryonic tissue, which may improve our understanding and assessment of human developmental toxicity risks in vivo.

In conclusion, we here provide a versatile and scalable platform for morphotoxicity screening on peri and early postimplantation stage human embryo models and demonstrate the importance of charting morphotoxicity with complimentary embryo models for a more accurate assessment of toxicity at a given developmental stage. This approach has the potential to advance toxicity assessment with embryo models, consequently reducing animal studies, mitigating the ethical constraints of using real human embryos, and redefining regulatory frameworks for assessing reproductive safety.

## 4 Materials and Methods

### 4.1 Cell Culture

H9 ESCs (WiCell, WA09) were cultured in mTESR1 medium (STEMCELL TECHNOLOGIES) and on 1% Geltrex‐coated tissue culture plates. REUS2 ESCs were cultured in NutriStem medium (Sartorius) and on 1% Vitronectin‐coated tissue culture plates. Both ESC lines were maintained in humidified incubators at 37°C and 5% CO_2_. ESCs were passaged every 5–7 days with TrypLE Express (Gibco), seeded at 5.000‐10.000 cells/cm^2^ and cultured in the presence of 10 µM Y‐27632 for 24 h after passaging.

Naïve iPSC O4/S17 (iPSCs from HDF75 fibroblasts, transduced with Oct4‐GFP and Sox17‐dTomato) were cultured in PXGL medium (Bredenkamp et al., 2019) on top of irradiated DR4 mouse embryonic fibroblasts (MEF; iPS core Erasmus Rotterdam). PXGL medium is N2B27 (DMEM/F12:Neurobasal 1:1, 1% N_2_ (made in‐house), 2% B27 (Gibco), 2 mM L‐glutamine (Gibco), 0.1 mM beta‐mercaptoethanol (Gibco) medium supplemented with 1 µM PD0325901, 2 µM XAV939, 2 µM Gö6983 and 10 ng/mL human LIF. Cells were dissociated every 3–4 days with Accutase for 3 to 5 min, spun down in DMEM/F12 (Sigma‐Aldrich) + 0.15% BSA V fraction (Gibco) for 3 min at 200 × *g* and resuspended in PXGL medium. The cell suspension was reseeded in 1:2 to 1:8 ratio on new wells with MEF monolayers. For the first 24 h of culture, 10 µM Y‐27632 was added. Cells were kept at 37°C in 5% O_2_, 5% CO_2_.

All cells were used for experiments after two passages post thawing at the earliest. No antibiotics were added to the cultures. Cells were not kept in culture beyond 60 passages. Cells were periodically tested for mycoplasma.

### 4.2 Microwell Preparation

For the generation of 3D structures from PSCs, 300 µm STATARRAY microwell plates from 300MICRONS were used (STATARRAYS MCA96‐16.224‐PS). Plates were washed with 100% ethanol to wet the surface. Thereafter the plates were incubated with 70% ethanol for at least 30 min to sterilize them. They were then washed at least thrice with PBS and incubated with a 1% (w/v) pluronic F108 solution for at least 1 h to prevent cell attachment. Afterwards they were washed once with DMEM and either stored for up to a week at 4°C or used immediately.

### 4.3 EPIC and AMNIC Culture

Microwells were prepared by removing the DMEM and adding 50 µL/well of mTeSR1 or NutriStem supplemented with 10 µM Y‐27632. The wells were preincubated in a humidified incubator at 37°C and 5% CO_2_. H9 ESCs or REUS2 were dissociated as described above, resuspended in mTeSR1 or NutriStem supplemented with 10 µM Y‐27632 and counted with Neubauer counting chambers. For EPICs, cells were seeded at 4.350 cells/well, which equals about 20 cells/microwell. For AMNICS, cells were seeded at 10.860 cells/well, which corresponds to 50 cells/microwells. Cells were left to settle in the microwells for 10–15 min in the incubator. For EPICs, the medium was then topped up to the final volume (250 µL/well). For AMNICs, undiluted Geltrex was added to cold NutriStem supplemented with Y‐27632 to a concentration of 0.5% Geltrex. Of this medium, 50 µL was added per well to reach a final concentration of 0.1% Geltrex in the microwells. Wells were then topped up to the final volume (250 µL/well). The cells were cultured at 37°C and 5% CO_2_. After 24 h, cells had formed aggregates and 150 µL/well was switched to plain mTeSR1 medium for the EPICs. For the AMNICs, 150 µL/well medium was changed to NutriStem supplemented with 16.7 ng/mL BMP4, for a final concentration of 10 ng/mL BMP4 in the microwells. Aggregates were cultured up to 72 h.

### 4.4 Blastoid Culture

One passage before blastoid culture, the naïve iPSCs were seeded on wells coated with 1% Geltrex (Gibco) to deplete the MEFs. After 3 days on Geltrex, cells were dissociated with TrypLE Express (Gibco) for 3–5 min and spun down in DMEM/F12 (Sigma‐Aldrich) + 0.15% BSA V fraction (Gibco) for 3 min at 200 × *g*. Cells were then resuspended in PALY medium [16], which was N2B27 medium supplemented with 2 µM PD0325901, 2 µM A83‐01, 10 µM Y‐27632 and 0.5 µM LPA. Cell counting was done manually using Neubauer counting chambers. Prior to cell seeding, DMEM was removed from microwells that were prepared as described above. The DMEM was replaced with 50 µL/well PALY medium to aid the even distribution of cells upon seeding. Cells were seeded at 20.000 cells/well, which corresponds to 100 cells/microwell on average. Plates were moved to an incubator (37°C, 5% CO_2_) for 10–15 min to allow cells to collect in the bottoms of the microwells. After the cells had settled, the medium was topped up to the final volume (250 µL/well). PALY medium was refreshed after 24 h. After 48 h, medium was replaced with N2B27 supplemented with 0.5 µM A83‐01.

### 4.5 Compound Library Preparation

All compounds were dissolved in either sterile DMSO or water, as indicated in Supplementary Table S1, except for folic acid, which was dissolved in 1 M NaOH. Stocks were then diluted in DMEM to 5X the final concentration and pipetted in a master plate for easy handling during the experiment. Master plates were stored at −20°C and thawed at 4°C prior to use. Water controls were done with 20%, 2.5%, and 0.25% final concentrations, DMSO controls with 2%, 1%, and 0.5%, and NaOH controls of 2%, 0.2%, and 0.02%, to cover the full range of solvent concentrations used in this set‐up. The library was composed to contain both positive and negative references, as well as several unreported compounds.

### 4.6 Toxicity Screening

All in vitro embryo models used for toxicity screening were exposed to the compound library for 24 h. Briefly, each embryo model was cultured up to 48 h in the microwells according to their normal culture protocol. At 48 h, 150 µL of medium was removed and replaced with 100 µL of culture medium according to the embryo model's culture protocol and 50 µL of prediluted compounds. After 24 h of continued culture, all conditions were fixed by washing twice with PBS, followed by incubation of 15 min in 4% PFA (ThermoFisher Scientific). Structures were washed three times with PBS and incubated for 30 min with 0.25% Triton‐X (Sigma–Aldrich) supplemented with Hoechst (1 µg/mL, ThermoFisher Scientific) and phalloidinAF568 (1:150, ThermoFisher Scientific) at room temperature. Structures were washed twice with PBS and stored at 4°C.

### 4.7 Immunofluorescence Staining

For immunofluorescence staining, samples were were fixed by washing twice with PBS, followed by incubation of 15 min in 4% PFA (ThermoFisher Scientific). Structures were washed three times with PBS and incubated for 30 min with 0.25% Triton‐X (Sigma–Aldrich), followed by one hour incubation with PBS + 1% BSA (V fraction (Gibco)). Afterwards, samples were incubated with primary antibodies in PBS +0.1% BSA + 0.125% Triton‐X at 4°C overnight. Samples were washed four times with PBS and incubated with secondary antibodies (AlexaFluor, ThermoFisher) and Hoechst (1 µg/mL) for 1–1.5 h at room temperature the following day. Finally, samples were washed thrice and stored in PBS at 4°C.

Antibodies used were OCT4 (R&D Systems, AF1759), GATA3 (Abcam, ab199428), TFAP2A (Santa Cruz, sc‐12726), and cleaved Caspase3 (Cell Signaling Technology, 9661S).

### 4.8 Microscopy

Fixed samples were imaged with a Nikon Eclipse Ti‐E spinning disk confocal imaging microscope (Japan) equipped with a Prime 95B sCMOS camera, Lumencor Spectra X Light source and a CrestOptics X‐Light V2 spinning disk unit with a pinhole size of 70 µm. The acquisition was performed with a 10x objective and automated using the NIS Elements JOBS module. For the blastoids, Z‐stack montages were acquired with 7 focal planes spanning 200 µm. These were converted to focused z‐projections using the EDF function and the NIS Elements GA3 Batch module.

### 4.9 Image Analysis

Automated image analysis was performed using CellProfiler 4.2.6 and CellProfiler Analyst 3.0.4 [31, 58]. The CellProfiler pipelines for all three embryo models were constructed according to the following outline: Using Rescale Intensity, Gaussian filter and, in the case of blastoids, closing modules, images were prepared for object detection. The DAPI/Hoechst channel was used to identify primary objects. Measurement modules for AreaShape, Intensity, Intensity Distribution and Texture were applied to the primary objects. For EPICs and AMNICs, this data was loaded into CellProfiler Analyst. In Analyst, the Classifier tool was used to conduct training sessions on the data to derive rules using the Fast Gentle Boosting model. For this training, at least between 8 and 10 images of whole 96‐wp wells from one of the screening plates were selected per embryo model. Each image contained up to 169 AMNICs or EPICs. The images were selected to represent a mix of all possible outcomes (intact, disrupted morphology and disintegrated structures). From these images between roughly 40–60 structures were manually classified as either intact or not intact (approximately 30 structures per class) in Classifier. This data was then used by CellProfiler Analyst to derive rules for distinguishing intact EPICs or AMNICs. The yield output was validated by comparison to manual counting (percentage of cavitated structures per 96wp well).

### 4.10 Statistical Analysis

Statistical analysis was performed using Excel and GraphPad Prism 8.0.2. To provide a full yet insightful overview of the data set, all absolute values were normalized to the most appropriate control. For compounds in DMSO‐dissolved compounds, this meant that almost all conditions were normalized to the low DMSO control, as they were all diluted to less than 0.1% DMSO in the final concentration the embryo models were exposed to. The only exception is aspirin at a high concentration, where 2% DMSO was present. For water‐dissolved compounds, they were generally normalized to the low water control at low and medium concentrations. Several compounds dissolved in water contained more than 0.25% water their highest concentration. These conditions were therefore normalized to the medium water control of 2.5%. Only thiamine at its highest concentration contained 20% water and was therefore compared with the high concentration water control. Folic acid concentrations were matched with the NaOH control concentrations and thus were normalized to them.

Embryo model yields were analyzed using one‐way ANOVA followed by Dunnett's post hoc test, which provided appropriate comparison of multiple treatment conditions to shared controls while limiting the accumulation of false‐positive errors across the comparisons. Dunnett's test was applied because the yield data met the assumptions of normality and homoscedasticity. Projected areas of the embryo models were analyzed using two‐way ANOVA to evaluate the effects of compounds and their concentration. Selected pairwise comparisons of compound conditions with their relevant control conditions were subsequently conducted using Tamhane's T2 post hoc test. As Brown–Forsythe tests indicated that several projected area datasets violated the assumption of homoscedasticity, the Tamhane's T2 test was selected. For consistency across all projected area comparisons, Tamhane's T2 was applied to all projected area datasets irrespective of individual homoscedasticity test outcomes.

## Funding

The authors acknowledge financial support from Maastrichts Universitair Medisch Centrum+ (MUMC+), from Stichting De Weijerhorst, from NWO (Open Competition ENW XS), and from the Dutch Province of Limburg (program “Limburg INvesteert in haar Kenniseconomie/LINK”).

## Conflicts of Interest

S.G. is one of the founders and shareholders of 300MICRONS GmbH.

## Use of Generative AI and AI‐Assisted Technologies in the Writing Process

Generative AI tools (ChatGPT 4.0 and Perplexity) were used for the proofreading and reference checking of the introduction section.

## Supporting information




**Supporting File 1**: adhm71613‐sup‐0001‐SuppMat.docx.

**Supporting File 2**: adhm71613‐sup‐0002‐Data.zip.

## Data Availability

The data that support the findings of this study are openly available in Zenodo at https://doi.org/10.5281/zenodo.21063319, reference number 21063319 [59].
