# Supplementary material for: Charting Morphotoxicity With Complementary Embryo Models
Source: Adv Healthc Mater. 2026 Aug 27;15(35):e03634. doi: 10.1002/adhm.202503634 (PMC13586646; doi:10.1002/adhm.202503634)
Supplement: Supplementary file 1 [file ADHM-15-0-s001.docx]

# Supplementary information

Charting morphotoxicity with complementary embryo models

Dorian G. Luijkx^1^, Pauline L. Balder^1,2#^, Romy M.L. Verhagen^1#^, Vinidhra Shankar^1^, Barry Jutten^1^, Pinak Samal^1^, Timo Rademakers^1,3,4^, Clemens A. van Blitterswijk^1^, Stefan Giselbrecht^1^*, Erik J. Vrij^1,2,^*

1. MERLN Institute for Technology-Inspired Regenerative Medicine, Department for Cell-inspired Tissue Engineering, Maastricht University, Universiteitssingel 40, 6229ET Maastricht, The Netherlands
2. MERLN Institute for Technology-Inspired Regenerative Medicine, Department for Complex Tissue Regeneration, Maastricht University, Universiteitssingel 40, 6229ET Maastricht, The Netherlands
3. MERLN Institute for Technology-Inspired Regenerative Medicine, Department for Instructive Biomaterials Engineering, Maastricht University, Universiteitssingel 40, 6229ET Maastricht, The Netherlands
4. GROW Research Institute for Oncology and Reproduction, Maastricht University, Universiteitssingel 40, 6229ET Maastricht, the Netherlands

#These authors contributed equally to this work

*These authors contributed equally to this work

Corresponding author: [e.vrij@maastrichtuniversity.nl](mailto:e.vrij@maastrichtuniversity.nl), [s.giselbrecht@maastrichtuniversity.nl](mailto:s.giselbrecht@maastrichtuniversity.nl)

Supplementary Figure 1. Quantification of immunofluorescence intensity of OCT4 (epiblast marker) and TFAP2α (amnion marker) in EPICs and AMNICs. Intensities were measured in 30 co-stained EPICs and AMNICs.

Supplementary Figure 2. Example merged images of brightfield, Hoechst (blue) and phalloidin (orange) of EPICs’ formation in a 96 microtiter plate. A) Intact EPICs with a lumen, B) disintegrated EPICs and C) EPICs with a disrupted morphology (no lumen). Scale bars depict 500 µm.

Supplementary Figure 3. Validation of blastoid screening. A-C) Manual validation of automated yield measurements performed on one of the replicate plates of the blastoid screen, shown as the percentage of cavitated structures out of all cell aggregates, N=1. A) Yields in conditions with either water- or NaOH-dissolved compounds, B) Yields in conditions with DMSO-dissolved compounds and C) Correlation scatterplot of automated versus manual yield measurements. D-E) Comparison of vehicle controls with medium control, where vehicle control concentrations from low to high were 0.5%, 1% and 2% for DMSO, 0.25%, 2.5% and 20% for H2O and 0.02%, 0.2% and 2% for NaOH, n=1. D) Yields shown as percentages of cavitated structures out of all cell aggregates and E) Area of blastoids measured in µm^2, sample size dependent on the number of blastoids per condition. L indicates the low, M the medium and H the high concentration tested for each compound. Error bars in D and E indicate standard error.

Supplementary Figure 4. Validation of AMNICs screening. A-C) Manual validation of automated yield measurements performed on one of the replicate plates of the AMNIC screen, shown as the percentage of cavitated structures out of all cell aggregates, N=1. A) Yields in conditions with either water- or NaOH-dissolved compounds, B) Yields in conditions with DMSO-dissolved compounds and C) Correlation scatterplot of automated versus manual yield measurements. D-E) Comparison of vehicle controls with medium control, where vehicle control concentrations from low to high were 0.5%, 1% and 2% for DMSO, 0.25%, 2.5% and 20% for H2O and 0.02%, 0.2% and 2% for NaOH, n=1. D) Yields shown as percentages of cavitated structures out of all cell aggregates and E) Area of AMNICs measured in µm^2, sample size dependent on the number of AMNICs per condition. L indicates the low, M the medium and H the high concentration tested for each compound. Error bars in D and E indicate standard error.

Supplementary Figure 5. Validation of EPICs screening. A-C) Manual validation of automated yield measurements performed on one of the replicate plates of the EPIC screen, shown as the percentage of cavitated structures out of all cell aggregates, N=1. A) Yields in conditions with either water- or NaOH-dissolved compounds, B) Yields in conditions with DMSO-dissolved compounds and C) Correlation scatterplot of automated versus manual yield measurements. D-E) Comparison of vehicle controls with medium control, where vehicle control concentrations from low to high were 0.5%, 1% and 2% for DMSO, 0.25%, 2.5% and 20% for H2O and 0.02%, 0.2% and 2% for NaOH, n=1. D) Yields shown as percentages of cavitated structures out of all cell aggregates and E) Area of EPICs measured in µm^2, sample size dependent on the number of EPICs per condition. L indicates the low, M the medium and H the high concentration tested for each compound. Error bars in D and E indicate standard error.

Supplementary Figure 6. Immunofluorescence staining of cleaved Caspase3 in AMNICs, blastoids and EPICs to illustrate a spectrum of toxicity effects. Ubiquitous cleaved Caspase 3, a marker for apoptosis, is indicative of cytotoxicity (top images), while developmental toxicity may disrupt morphologies without causing cell death (bottom images).

Supplementary Table 1. Overview of compounds, their stock concentrations, the highest concentration tested in this study, the minimal dilution factor based on the highest concentration tested, the subsequent concentration of the vehicle at the high concentration, the vehicle per compound (DMSO, water or NaOH) and for high, medium and low concentrations of each compound tested in the screen, the vehicle control concentration (low, medium or high) that each condition was normalized to.

| **compound** | **stock**  **(in mM)** | **[high] (in uM)** | **dilution factor** | **final% for [high]** | **vehicle** | **[high]** | **[medium]** | **[low]** |
| --- | --- | --- | --- | --- | --- | --- | --- | --- |
| 2,4,6-triiodophenol | 200 | 100 | 800 | 0,1% | DMSO | low | low | low |
| 5-Fluorouracil | 76,88 | 500 | 153,8 | 0,7% | H_2_O | high | medium | low |
| Acetazolamide | 200 | 121 | 1652,9 | 0,06% | DMSO | low | low | low |
| Ascorbic acid | 432,1 | 1136 | 380,4 | 0,3% | H_2_O | medium | low | low |
| Aspirin | 277 | 5000 | 55,4 | 1,8% | DMSO | high | low | low |
| Benztropine mesylate | 200 | 200 | 1000 | 0,1% | H_2_O | medium | low | low |
| Bosentan | 100 | 40 | 2500 | 0,04% | DMSO | low | low | low |
| Busulfan | 100 | 40,6 | 2463,1 | 0,04% | DMSO | low | low | low |
| Caffeine | 100 | 22,66 | 4413,1 | 0,02% | H_2_O | low | low | low |
| Carbamazepine | 200 | 423,25 | 472,5 | 0,2% | DMSO | low | low | low |
| Cytosine arabinoside | 200 | 5 | 40000 | 0,003% | DMSO | low | low | low |
| Dexamethasone | 200 | 1 | 200000 | 0,0005% | DMSO | low | low | low |
| Diphenhydramine | 3355 | 784 | 4279,3 | 0,02% | DMSO | low | low | low |
| Folic Acid | 113,3 | 2265,5 | 50,0 | 2,0% | NaOH | high | medium | low |
| Glycolic acid | 1000 | 5000 | 4000 | 0,03% | H_2_O | low | low | low |
| Hydroxyurea | 1000 | 100 | 10000 | 0,01% | DMSO | low | low | low |
| Ibuprofen | 200 | 250 | 800 | 0,1% | DMSO | low | low | low |
| Isoniazid | 365 | 7291,9 | 50,1 | 2,0% | H_2_O | medium | low | low |
| Methoxyacetic acid | 1000 | 5000 | 200 | 0,5% | H_2_O | medium | low | low |
| Phenytoin | 200 | 20 | 10000 | 0,01% | DMSO | low | low | low |
| Phthalic acid | 200 | 100 | 2000 | 0,05% | DMSO | low | low | low |
| Propafenone hydrochloride | 8,27 | 30 | 275,7 | 0,4% | DMSO | medium | low | low |
| Saquinavir mesylate | 20 | 10 | 2000 | 0,05% | DMSO | low | low | low |
| Thalidomide | 200 | 100 | 2000 | 0,05% | DMSO | low | low | low |
| Thiamine | 200 | 37685,4 | 3,9 | 25,5% | H_2_O | high | medium | low |
| trans Retinoic Acid | 0,1 | 0,1 | 1000 | 0,1% | DMSO | low | low | low |
| Valproic Acid | 200 | 1000 | 200 | 0,5% | DMSO | low | low | low |

Supplementary Table 2. Normalized area averages for each condition across the three models (blastoids, AMNICs and EPICs).

| **compound** | **[c]** | **EPICs** | **AMNICs** | **blastoids** |
| --- | --- | --- | --- | --- |
| 2,4,6-triiodophenol | low | 0,983 | 1,140 | 1,046 |
| 2,4,6-triiodophenol | medium | 0,946 | 1,142 | 0,868 |
| 2,4,6-triiodophenol | high | 1,147 | 1,062 | 0,644 |
| Acetazolamide | low | 1,179 | 1,042 | 0,917 |
| Acetazolamide | medium | 0,964 | 1,108 | 1,091 |
| Acetazolamide | high | 1,258 | 1,066 | 1,020 |
| Aspirin | low | 1,234 | 1,149 | 1,153 |
| Aspirin | medium | 1,111 | 1,143 | 1,205 |
| Aspirin | high | 0,759 | 0,856 | 0,731 |
| Bosentan | low | 0,425 | 1,017 | 1,007 |
| Bosentan | medium | 1,319 | 1,090 | 1,183 |
| Bosentan | high | 1,151 | 1,036 | 1,199 |
| Busulfan | low | 0,903 | 1,023 | 1,112 |
| Busulfan | medium | 1,229 | 1,093 | 1,122 |
| Busulfan | high | 0,863 | 1,090 | 1,097 |
| Carbamazepine | low | 1,418 | 1,023 | 1,070 |
| Carbamazepine | medium | 1,294 | 1,004 | 1,064 |
| Carbamazepine | high | 0,837 | 0,923 | 0,830 |
| Cytosine arabinoside | low | 1,139 | 0,988 | 1,271 |
| Cytosine arabinoside | medium | 0,832 | 0,812 | 1,188 |
| Cytosine arabinoside | high | 0,021 | 1,154 | 1,236 |
| Dexamethasone | low | 1,047 | 1,003 | 0,931 |
| Dexamethasone | medium | 0,704 | 1,055 | 0,848 |
| Dexamethasone | high | 0,825 | 0,816 | 1,129 |
| Diphenhydramine | low | 1,233 | 1,105 | 0,853 |
| Diphenhydramine | medium | 0,904 | 0,949 | 0,993 |
| Diphenhydramine | high | 0,240 | 0,812 | 0,417 |
| Hydroxyurea | low | 0,486 | 0,955 | 1,093 |
| Hydroxyurea | medium | 1,119 | 0,975 | 1,011 |
| Hydroxyurea | high | 1,372 | 1,004 | 0,899 |
| Ibuprofen | low | 1,174 | 1,106 | 0,992 |
| Ibuprofen | medium | 0,946 | 1,027 | 1,115 |
| Ibuprofen | high | 1,308 | 1,060 | 1,140 |
| Phenytoin | low | 1,058 | 1,128 | 1,055 |
| Phenytoin | medium | 1,256 | 1,044 | 1,170 |
| Phenytoin | high | 0,817 | 0,982 | 1,126 |
| Phthalic acid | low | 1,594 | 1,070 | 1,056 |
| Phthalic acid | medium | 1,034 | 0,942 | 1,002 |
| Phthalic acid | high | 0,753 | 0,974 | 1,011 |
| Propafenone hydrochloride | low | 0,555 | 0,658 | 0,334 |
| Propafenone hydrochloride | medium | 1,186 | 1,141 | 0,948 |
| Propafenone hydrochloride | high | 1,064 | 1,125 | 0,899 |
| Saquinavir mesylate | low | 1,079 | 1,167 | 1,022 |
| Saquinavir mesylate | medium | 0,869 | 1,072 | 0,832 |
| Saquinavir mesylate | high | 0,648 | 1,073 | 0,376 |
| Thalidomide | low | 1,385 | 1,124 | 0,917 |
| Thalidomide | medium | 1,183 | 1,071 | 1,038 |
| Thalidomide | high | 0,897 | 1,103 | 1,016 |
| trans Retinoic Acid | low | 1,377 | 1,040 | 0,925 |
| trans Retinoic Acid | medium | 1,521 | 1,018 | 0,611 |
| trans Retinoic Acid | high | 1,350 | 0,994 | 0,913 |
| Valproic Acid | low | 1,407 | 1,041 | 0,923 |
| Valproic Acid | medium | 0,831 | 1,029 | 1,203 |
| Valproic Acid | high | 0,466 | 1,027 | 1,195 |
| 5-Fluorouracil | low | 0,000 | 1,042 | 0,875 |
| 5-Fluorouracil | medium | 0,000 | 1,048 | 0,779 |
| 5-Fluorouracil | high | 0,000 | 1,112 | 0,547 |
| Ascorbic acid | low | 1,014 | 0,981 | 0,948 |
| Ascorbic acid | medium | 0,937 | 0,959 | 0,839 |
| Ascorbic acid | high | 0,821 | 0,927 | 0,900 |
| Benztropine mesylate | low | 1,263 | 1,004 | 0,831 |
| Benztropine mesylate | medium | 0,147 | 0,713 | 0,352 |
| Benztropine mesylate | high | 0,000 | 0,171 | 0,618 |
| Caffeine | low | 0,868 | 0,935 | 0,914 |
| Caffeine | medium | 1,084 | 0,947 | 0,948 |
| Caffeine | high | 1,232 | 0,956 | 0,865 |
| Folic Acid | low | 0,907 | 1,015 | 0,919 |
| Folic Acid | medium | 0,745 | 0,707 | 1,082 |
| Folic Acid | high | 1,307 | 0,921 | 1,250 |
| Glycolic acid | low | 1,082 | 1,025 | 0,931 |
| Glycolic acid | medium | 1,052 | 1,053 | 0,991 |
| Glycolic acid | high | 1,002 | 1,034 | 1,190 |
| Isoniazid | low | 1,152 | 0,997 | 0,897 |
| Isoniazid | medium | 1,034 | 0,984 | 0,780 |
| Isoniazid | high | 0,739 | 1,053 | 0,921 |
| Methoxyacetic acid | low | 0,410 | 0,984 | 1,026 |
| Methoxyacetic acid | medium | 0,293 | 0,830 | 0,331 |
| Methoxyacetic acid | high | 0,049 | 0,622 | 0,405 |
| Thiamine | low | 1,080 | 1,036 | 0,929 |
| Thiamine | medium | 0,637 | 1,048 | 0,902 |
| Thiamine | high | 1,048 | 0,912 | 0,960 |

Supplementary Data 1. CellProfiler pipeline file for automated analysis of blastoids in microwell images.

Supplementary Data 2. CellProfiler pipeline file for automated analysis of AMNICs in microwell images.

Supplementary Data 3. CellProfiler Analyst-derived rules for the filtering of intact AMNICs.

Supplementary Data 4. CellProfiler pipeline file for automated analysis of EPICs in microwell images.

Supplementary Data 5. CellProfiler Analyst-derived rules for the filtering of intact EPICs.
